# Evaluating Machine Learning-Based MRI Reconstruction Using Digital Image Quality Phantoms

**DOI:** 10.3390/bioengineering11060614

**Published:** 2024-06-15

**Authors:** Fei Tan, Jana G. Delfino, Rongping Zeng

**Affiliations:** Division of Imaging, Diagnostics and Software Reliability (DIDSR), Office of Science and Engineering Laboratories (OSEL), Center for Devices and Radiological Health (CDRH), U.S. Food and Drug Administration (U.S. FDA), Silver Spring, MD 20993, USA; fei.tan@fda.hhs.gov (F.T.); jana.delfino@fda.hhs.gov (J.G.D.)

**Keywords:** machine learning, MRI reconstruction, automated image quality evaluation, digital image quality phantom, image resolution, low-contrast detectability, digital reference object

## Abstract

Quantitative and objective evaluation tools are essential for assessing the performance of machine learning (ML)-based magnetic resonance imaging (MRI) reconstruction methods. However, the commonly used fidelity metrics, such as mean squared error (MSE), structural similarity (SSIM), and peak signal-to-noise ratio (PSNR), often fail to capture fundamental and clinically relevant MR image quality aspects. To address this, we propose evaluation of ML-based MRI reconstruction using digital image quality phantoms and automated evaluation methods. Our phantoms are based upon the American College of Radiology (ACR) large physical phantom but created in k-space to simulate their MR images, and they can vary in object size, signal-to-noise ratio, resolution, and image contrast. Our evaluation pipeline incorporates evaluation metrics of geometric accuracy, intensity uniformity, percentage ghosting, sharpness, signal-to-noise ratio, resolution, and low-contrast detectability. We demonstrate the utility of our proposed pipeline by assessing an example ML-based reconstruction model across various training and testing scenarios. The performance results indicate that training data acquired with a lower undersampling factor and coils of larger anatomical coverage yield a better performing model. The comprehensive and standardized pipeline introduced in this study can help to facilitate a better understanding of the performance and guide future development and advancement of ML-based reconstruction algorithms.

## 1. Introduction

Machine learning techniques are widely investigated for magnetic resonance imaging (MRI) reconstruction, addressing challenges such as undersampled k-space completion, image artifact removal, and direct transform from k-space to image domain [1]. Algorithm developers often evaluate image performance based on computer vision metrics such as root mean squared error (RMSE), peak signal-to-noise ratio (PSNR), and structural similarity (SSIM) [2,3,4]. Although these metrics are convenient to calculate, they often fall short in capturing critical physical MR image quality metrics, such as resolution, homogeneity, and low-contrast detectability, which are more clinically relevant for medical imaging applications.

MR image quality phantoms with various testing objects and image evaluation methods have been developed by the American College of Radiology (ACR) [5] to offer standardized benchmarks to assess these physical image quality metrics across multiple scanners and imaging centers [6,7]. Unfortunately, these evaluation methods heavily rely on human interpretation, increasing the cost and measurement variability. Automated evaluation methods without the need for human intervention are desired to improve objectivity and reduce evaluation time. Some automated quantification algorithms for physical ACR phantoms exist [8,9,10,11] and demonstrate a reasonable correlation with manual measurements. However, not all of the physical image quality metrics specified in ACR phantom test guidance are included, especially the low-contrast detectability metric. In addition, for evaluating machine learning algorithms, large imaging datasets are needed to test performance generalizability in various data acquisition protocols and thus it may not be practical to physically scan phantoms hundreds of times. We address this need by developing a comprehensive MR image quality evaluation pipeline. Our evaluation pipeline includes both the simulated MR images of the test objects as well as fully automated evaluation algorithms for a comprehensive set of relevant physical image quality metrics, including geometric accuracy, intensity uniformity, percentage ghosting, sharpness, signal-to-noise ratio, high-contrast resolution, and low-contrast detectability. It offers a flexible and scalable alternative for evaluating machine learning reconstruction algorithms because of its ability to generate a vast amount of data with diverse characteristics and without the need for human interpretation.

Existing MR phantom simulation algorithms [12,13] that create geometric or anthropomorphic digital phantoms are typically designed in a discrete image space that does not model physically continuous objects. This can result in loss of resolution in object models, degrading the evaluation accuracy. A recent advancement in using digital reference objects (DROs) to evaluate MR image resolution [14] has shown promise for evaluating machine learning MR image denoising networks. This study created digital image resolution phantoms to assess high-contrast image resolution before and after applying a bias-free denoising convolutional neural network (BF-DnCNN) [15]. Yet, this study focused on only one metric (image resolution) and thus is limited in scope. Built upon the DRO work, we also mathematically model continuous test objects in the k-space domain. We further extend the design to include multiple test phantoms to enable the evaluation of a broader set of image quality metrics, inspired by the established American College of Radiology (ACR) physical MR phantoms [5] and National Electrical Manufacturers Association (NEMA) standards [16,17].

Using the Automated Transform by Manifold Approximation (AUTOMAP) [2] MR reconstruction network as an example, we show our approach to be a versatile tool for assessing machine learning-based MR reconstruction methods and optimizing model development. We use our evaluation pipeline to study the impact of various training and testing data scenarios on the AUTOMAP reconstruction network.

Overall, the main research contribution of our work is the introduction of a standardized pipeline that includes digital image quality phantoms and automated image quality evaluation metrics, bridging a critical gap in the evaluation of machine learning-based MR reconstruction. We create the digital phantoms in continuous k-space to better simulate the MR acquisition process. By leveraging digital phantoms and automated evaluation algorithms, researchers and clinicians can gain deeper insight into the performance of MR reconstruction algorithms, which can guide future improvements in these MR reconstruction algorithms.

The structure of the paper is organized as follows: Section 2 describes the image quality assessment pipeline, including digital phantom creation and the automated evaluation metrics, and introduces the example ML reconstruction network with the training and testing dataset composition and experimental designs as a use case for this assessment pipeline. Section 3 presents the performance results of the example reconstruction network across various training and testing scenarios considered in our experiment design. Section 4 discusses the findings from the experiments, limitations, and future work, followed by conclusions in Section 5.

## 2. Materials and Methods

In this section, we introduce the creation of the digital phantoms and the definitions of the automated evaluation metrics. We also describe the example reconstruction network and the datasets used for training. Additionally, we conduct experiments under various training or testing scenarios to demonstrate the utility of our image quality evaluation pipeline for analyzing the impact of training and testing data on reconstruction network performance.

### 2.1. Digital Phantom Creation

**Figure 1:**
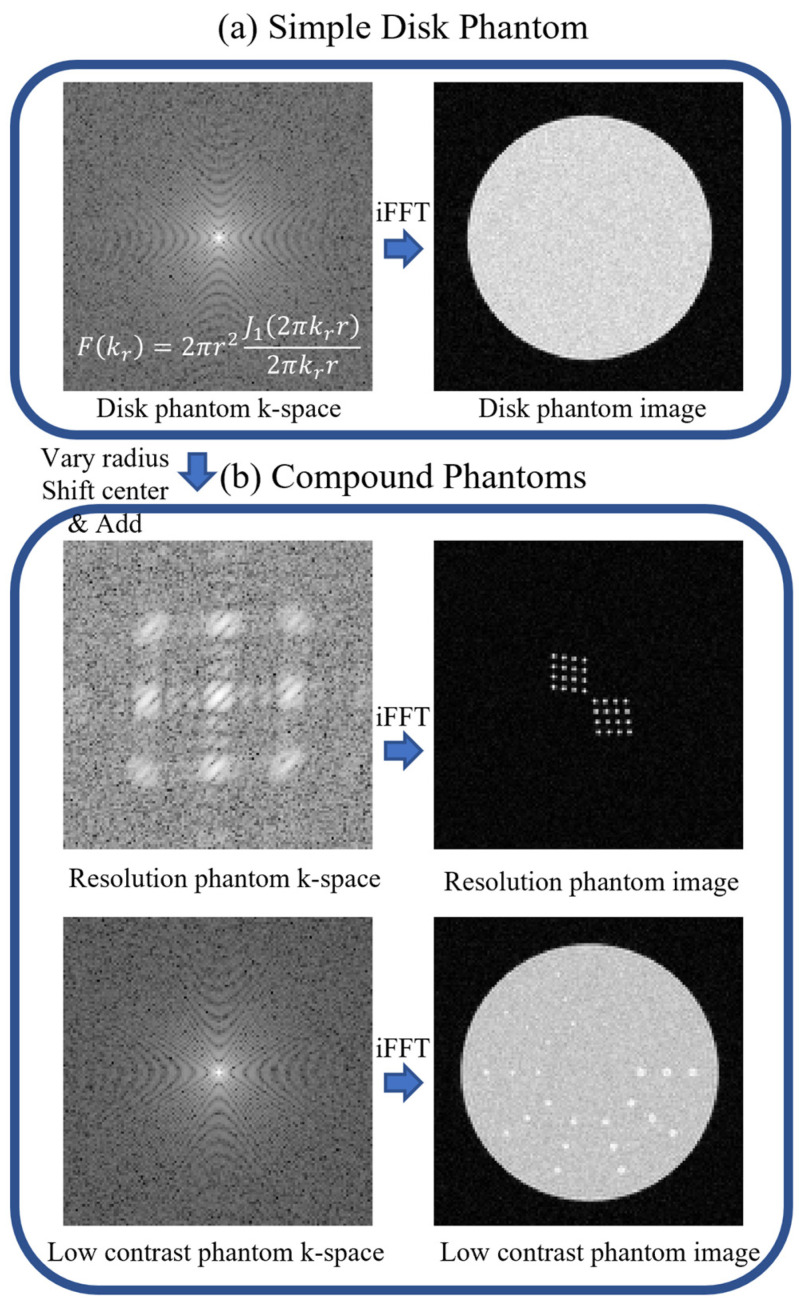
Pipeline for creating (**a**) simple disk phantom and (**b**) compound phantoms, including a resolution phantom and a low-contrast phantom. All of the phantoms are created in k-space (2D frequency domain) using its mathematical definition and Fourier theorem. The phantoms in image space are calculated by simple inverse Fast Fourier transform (iFFT).

In accordance with the physical phantoms recommended by American College of Radiology (ACR) large phantom guidance [5], we developed digital phantoms for the evaluation of image quality (IQ) in machine learning-based MRI reconstruction. Our IQ phantoms are composed of single or multiple geometrically defined disk objects, the Fourier transforms of which in k-space can be described by analytical functions. As depicted in Figure 1, these digital phantoms are then sampled in k-space and inverse Fast Fourier Transform (iFFT) is applied to obtain the corresponding MR images [14]. Such phantom image creation approach better approximates the MRI acquisition process that scans a continuous object to form a discretized image. Our design contains three types of phantoms.

1. Simple Disk Phantom:

A simple disk phantom, described by the set of parameters $\left\{R,I,x_{c},y_{c}\right\},$ corresponding to its radius, intensity, and *x*-axis and *y*-axis center coordinates, is constructed using its continuous Fourier k-space expression $F_{disk}$, defined by the Jinc function, as follows:

(1) $$F_{disk}\left(k_{r}\right|R,I,x_{c},y_{c})=Jinc\left(k_{r}\right|R)\times I\times e^{-2\pi i(x_{c}k_{x}+y_{c}k_{y})},\;\mathrm{with}\;Jinc\left(k_{r}|R\right)=2\pi R^{2}\frac{J_{1}\left(2\pi Rk_{r}\right)}{2\pi Rk_{r}},$$

where $J_{1}(\cdot )$ denotes the 1st order Bessel function of the first kind, and $k_{x}\;and\;\;k_{y}$ are the coordinates in k-space, with $k_{r}=\sqrt{k_{x}^{2}+k_{y}^{2}}$. As shown in Equation (1), an off-center disk in image space corresponds to a phase shift of $e^{-2\pi i(x_{c}k_{x}+y_{c}k_{y})}$ in k-space. A complex Gaussian noise map with zero mean and standard deviation of σ, denoted by $\psi_{0,\sigma }(k_{x},\;k_{y})$, can be added to model the additive MR acquisition noise. The continuous noisy k-space function can then be sampled with an interval of $\delta k_{x}=\delta k_{y}=\frac{1}{FOV}$ to create discretized k-space MR data, where FOV represents the size of the MR image field of view (FOV).

The parameters for the simple disk phantom, such as radius, center, image intensity, noise level, and FOV, can be varied. Note that the parameters (except image intensity) are in physical units instead of the number of pixels. A simple disk phantom can be used to evaluate the geometric accuracy, intensity uniformity, percentage ghosting, sharpness, and signal-to-noise ratio, as described in Section 2.2.

2. Resolution Phantom

The resolution phantom is a compound phantom constructed by superimposing two 4 × 4 arrays of small disk phantoms to quantify the image resolution in both directions. Each row of disks is shifted by ½ radius to account for the partial volume effect. Parameters such as the number of disks per row, disk size, and noise level can be varied to customize the resolution phantom.

The formation of the compound phantom involves adding the k-space of disks of various sizes and at multiple locations. The equation governing this process is as follows:

(2) $$F_{compound}\left(k_{r}\right)=\sum_{i}F_{i}\left(k_{r}\right)$$

where $F_{compound}\left(k_{r}\right)$ represents the compound phantom function in k-space, and $F_{i}\left(k_{r}\right)$ denotes the k-space function of an individual disk as in Equation (1), with i being the disk number index. Similar to the simple disk phantom, complex Gaussian noise $\psi_{0,\sigma }(k_{x},k_{y})$ can be added to the compound phantom to model the additive MR acquisition noise.

3. Low-Contrast Phantom

The low-contrast phantom is also a compound phantom generated by superimposing small, low-intensity disks onto a large background disk. Following the ACR large phantom design, the disks are arranged in rows radiating from the center of a circle like spokes. The disks in each spoke have the same radius, but the radius gradually decreases from spoke to spoke. Parameters such as the number of spokes, number of disks per spoke, contrast, disk radius, and noise level can be adjusted.

For each category, we generated 600 noisy realizations based on power analysis with significance criterion *p* = 0.05 [18]. The default common parameters used were: FOV: 240 mm × 240 mm, matrix size: 128 × 128, disk intensity: 0.5, and two levels of standard deviation for complex Gaussian noise: 0.04 and 0.02, resulting in two signal-to-noise ratio (SNR) levels: 12.5 and 25. The significance criterion was selected according to [18]. The FOV was chosen to match that of two public datasets, and the matrix size was set based on the example ML reconstruction network input size. The background intensity level was set to be the mid-point of the normalized image intensity range of 0 to 1. Note that image intensity normalization was a preprocessing step used in AUTOMAP image reconstruction, as described later in Section 2.4.3. The noise levels were chosen to reproduce the noise level of the two public datasets we used. The example network and public datasets are described later in Section 2.3 and Section 2.4, respectively.

The phantom-specific parameters included: the radius of the disk phantom was equal to 95 mm; the radii of the resolution phantom ranged from 0.8 to 1.1 mm, corresponding to resolutions of 1.6 to 2.2 mm; and the low-contrast phantom had 10 spokes with 3 disks per spoke, with disk radii evenly spaced between 0.75 to 3.5 mm across the 10 spokes. The contrast was defined as the intensity ratio between the small disks and the background disk and was selected as 0.2. The disk phantom radius and the number of spokes and disk size of the low-contrast phantom were designed according to ACR phantom specifications. The radii range of the resolution phantom was selected to span across the designed pixel resolution of 1.875 mm. The intensity contrast of the low-contrast phantom was empirically chosen to ensure that the detection task was neither too simple nor too difficult.

The reference MR images of these digital phantoms were calculated from k-space using inverse Fast Fourier Transform (iFFT).

### 2.2. Evaluation Metrics

#### 2.2.1. Geometric Accuracy

**Figure 2:**
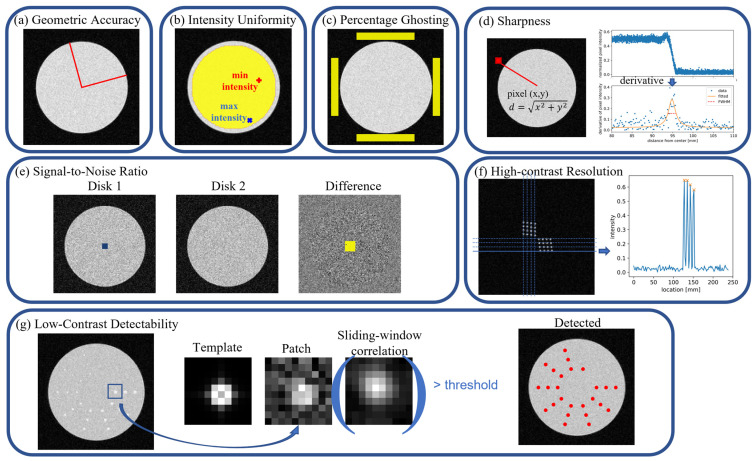
Illustration of image quality evaluation process. (**a**) Geometry accuracy is calculated by the maximum percentage radius error. (**b**) Intensity uniformity is defined by the percentage intensity uniformity within the large ROI of the disk. (**c**) Percentage ghosting is calculated using the average intensity of four background ROIs and a center foreground ROI. (**d**) Sharpness is measured by the full-width-half-maximum of the edge spread function. (**e**) SNR is calculated as the mean intensity divided by the standard deviation of the noise adjusted by a factor of $\sqrt{2}$. (**f**) Resolution is evaluated by peak separability. (**g**) Low-contrast detectability is quantified by the number of completely detected spokes. The red dots illustrate detected low-contrast disk locations using a template matching method.

Geometric accuracy was evaluated using the disk phantom (Figure 2a) [5]. A region growing algorithm detected the disk as an ellipse, measuring the radii of the major and minor axis of the ellipse, denoted as $R_{major}\;and\;\;R_{minor}$, which were then compared with the designed ground truth radius $R_{ground\;truth}$. Geometric accuracy was defined by the Maximum Percentage Error, as follows:

(3) $$Maximum\;Percentage\;Error=100\times \max\left(\left|\frac{R_{major}-R_{ground\;truth}}{R_{ground\;truth}}\right|,\left|\frac{R_{minor}-R_{ground\;truth}}{R_{ground\;truth}}\right|\right)$$

#### 2.2.2. Intensity Uniformity

Intensity uniformity was assessed using the disk phantom (Figure 2b) [5,16]. The disk region was eroded by 5 pixels to form a region of interest (ROI) that excluded the edges. The image was then convolved with a 3-by-3 low-pass filter, following the NEMA standard [16], to minimize the effect of noise. The maximum intensity value and minimum intensity value of the low-pass filtered image within the ROI are noted as $I_{max}$ and $I_{min}$, respectively. Intensity uniformity was defined as the Percent Intensity Uniformity, as follows:

(4) $$Percent\;Intensity\;Uniformity=100\times \left(1-\frac{I_{max}-I_{min}}{I_{max}+I_{min}}\right)$$

#### 2.2.3. Percentage Ghosting

Percentage ghosting quantifies the degree of ghosting artifact [5], assuming Cartesian sampling where undersampling and aliasing occur only in the phase-encoding direction (Figure 2c). Four rectangular regions at the top, bottom, left and right of the disk, centered 7N/16 pixels away from the disk center, with long and short edges N/2 and N/16 (N = matrix size, set to 128 in this study), were selected as background regions, while one circular region within the disk, same as in Section 2.2.2., was chosen as the object. The average intensities within these regions are noted as $\bar{I_{top}}$, $\bar{I_{bottom}}$, $\bar{I_{left}}$, $\bar{I_{right}}$, and $\bar{I_{disk}}$. The Ghosting Ratio was then calculated as follows:

(5) $$Ghosting\;Ratio=\left|\frac{\left(\bar{I_{top}}+\bar{I_{bottom}}\right)-\left(\bar{I_{left}}+\bar{I_{right}}\right)}{2\times \bar{I_{disk}}}\right|$$

#### 2.2.4. Sharpness

Sharpness assessment utilized the disk phantom and involved measuring the full-width-half-maximum (FWHM) of the edge spread function (ESF) (Figure 2d) following the method described in [19]. To achieve subpixel precision, the distance to the designed disk center was calculated for each pixel in the image. Then, intensity values were plotted against the distance to the disk center. The values were then resampled to a uniform distance grid of 1/10 of a pixel to obtain an estimation of the ESF. In cases of noisy images, curve fitting of the ESF to a sigmoid function was applied to enhance the robustness against image noise [20]. The derivative of this resampled ESF curve was then fitted to a Lorentzian function (Equation (6)) to enable parametric analysis. Four parameters, including the amplitude A, vertical shift B, full-width-half-maximum Γ, and horizontal shift $x_{0}$, were fitted from the data, and the FWHM of the resulting Lorentzian curve Γ served as the metric for image sharpness, expressed as follows:

(6) $$L\left(x|\;x_{0},\Gamma ,A,B\right)=A\times \frac{1}{\pi }\frac{\frac{\Gamma }{2}}{{\left(x-x_{0}\right)}^{2}+{\left(\frac{\Gamma }{2}\right)}^{2}}+B$$

We chose the Lorentzian function because it is a single peaked function resembling the shape of the derivative of ESF and the parameter Γ can be readily used as a metric for the image sharpness measure.

#### 2.2.5. Signal-to-Noise Ratio

Signal-to-noise ratio (SNR) was measured using a pair of disk phantoms (Figure 2e) [17,21]. Two disks with the same intensity but of different random noise were subtracted, and SNR was computed as the mean intensity of Disk 1, denoted by $I_{Disk_{1}}$ divided by the standard deviation of the difference image, denoted by $I_{Diff}$, multiplied by $\sqrt{2}$, as follows:

(7) $$SNR=\frac{mean\left(I_{Disk_{1}}\right)\sqrt{2}}{std(I_{Diff})}$$

#### 2.2.6. High-Contrast Resolution

High-contrast resolution is defined as the smallest distinguishable signal distance (Figure 2f) [5]. It was evaluated using the resolution phantom consisting of arrays of small disks with a range of diameters, as described in Section 2.1. Resolution was considered achieved at the diameter of the small disk if four distinct peaks were detected by the peak detection algorithm in at least one row. The peak detection algorithm identified all local maxima by comparing neighboring values, with the peak locations at least two radii apart and the peak height larger than a threshold calculated using Ostu’s method [22] according to the intensity distribution. A similar criterion was applied for perpendicular direction resolution.

#### 2.2.7. Low-Contrast Detectability

Low-contrast detectability was quantified by the number visible spokes in the low-contrast phantom. A spoke is defined as visible when all of the disks on the spoke are detected, according to ACR phantom test guidance [2]. We applied a template matching method to automatically determine whether a disk was detectable or not [23]. As illustrated in Figure 2g, a 10-by-10-pixel square patch was cropped around the center of a disk and correlated with a template designed to be the noiseless MR image of the same sized disk generated by the phantom creation process. The template was shifted and correlated with the patch to obtain a correlation map, referred to as sliding-window correlation. The resulting maximum value in the correlation map was then compared to a predetermined threshold. If the value was greater than the threshold, then the disk in the patch was determined to be detectable; otherwise, the disk was undetectable.

The threshold was determined through a pre-designed experiment as follows. For each disk size, we simulated 100 disk-present and 100 disk-absent 10-by-10-pixel MR images, sharing the same matrix size, noise standard deviation, and contrast as those disk patches cropped from the low-contrast phantom images. The noiseless template was sliding-window correlated with the 200 image patches, and the maximum value was recorded for each patch. The threshold was tuned to yield the highest disk-present and disk-absent classification accuracy for the 200 image patches. This threshold was then used toward the low-contrast detection algorithm for this specific disk size, noise level, and contrast, on all of the different AUTOMAP model reconstructed low-contrast phantom images. However, if the maximum classification accuracy value was below 0.70 in this experiment, the disk could not be reliably detected; thus, the spoke of the corresponding disk size in the low-contrast phantom images would be deemed undetectable without performing the detection process described above.

### 2.3. AUTOMAP Network

We demonstrated the utility of our evaluation pipeline with an MR image reconstruction network named Automated Transform by Manifold Approximation and Projection (AUTOMAP) [2]. AUTOMAP was designed to transform sensor domain data into the image space and has previously been adopted for low-field MRI reconstruction [24].

**Figure 3:**
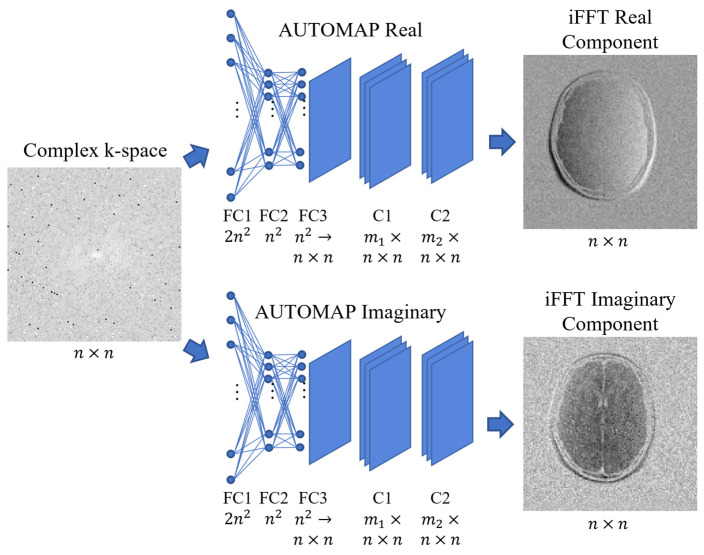
Example MR reconstruction network: AUTOMAP. Networks to estimate real and imaginary image components were trained separately. Both networks used the same hyperparameters as the original AUTOMAP structure. Distinct AUTOMAP networks were trained with M4Raw fully sampled and undersampled data and FastMRI fully sampled and undersampled data. Note that the digital phantom dataset was not included in training. The arrows suggest the flow of data.

As shown in Figure 3, we maintained the same structure and hyperparameters as the original AUTOMAP network [2]. The network contains three fully connected layers (FC) and two convolutional layers (C). The key parameters include an input matrix size of 128 × 128, 100 epochs with a batch size of 100, and a learning rate of 0.00002. Notably, we trained the network to separately estimate the real and imaginary components of the image and combined the output to construct the magnitude image.

The training of each network was conducted on a GPU machine equipped with an NVIDIA V100 GPU with 32 GB of memory. The training process typically took approximately 4 h for each network.

### 2.4. Training Data

We utilized two publicly available brain MR datasets, representing different MRI field strengths: one from clinical field strength (3T) and the other from low-field (0.3T) MR. These datasets were chosen because they provided complex k-space data. Note that only the brain MR data were included for training, and our digital phantom data were used purely for testing.

#### 2.4.1. M4Raw Dataset

The M4Raw dataset [25] is a publicly available dataset acquired at 0.3T. It comprises T1-weighted (T1w), T2-weighted (T2w), and fluid-attenuated inversion recovery (FLAIR) image contrast. The dataset contains training, validation, and test sets of 1024, 240, and 400 volumes, respectively. Each volume consists of 18 slices acquired with a four-channel head coil.

#### 2.4.2. Fast MRI Dataset

We used a subset of the FastMRI brain dataset [26,27] to match the number of training data in the M4Raw dataset. The selection criteria included a field strength at 3 Tesla and number of coils greater than 4, with data randomly selected from 4 coils. T1, T2, and FLAIR contrast were included and T1 post contrast were excluded to match the available contrast in the M4Raw dataset. Each FastMRI brain volume contains 2 to 16 slices. To align with the total number of training slices available from the M4Raw dataset and to balance the composition of T1w, T2w and FLAIR images, only 600 T2-weighted image volumes were selected.

#### 2.4.3. Preprocessing

To augment the total number of training data, 2D axial slices from each coil were treated as separate training images. This resulted in 73,728 training images for the M4raw- and 71,664 for FastMRI-trained networks. Preprocessing steps included cropping the central 128 × 128 matrix of the k-space, applying random phase shifts in k-space for image shifting to promote translational invariance, and normalizing both k-space and image space by the maximum image intensity. The reference image was obtained by computing the inverse Fourier transform.

Two sets of inputs were prepared for each dataset: one with fully sampled k-space and the other with k-space undersampled by a factor of two. Variable density undersampling with a center of 30% of fully sampled data was employed in the latter case and the k-space lines were only undersampled in the phase-encoding direction [26], which is the horizontal direction in this paper. The unsampled lines were zero filled to maintain the same matrix size.

### 2.5. Test Set

Two types of test datasets were utilized in our evaluation. The brain test set, comprising 28,800 images from M4Raw and 16,752 images from FastMRI test sets, was utilized to compute conventional machine learning metrics such as the structural similarity index (SSIM), peak signal-to-noise ratio (PSNR), and root mean squared error (RMSE). These metrics reflect the global similarity and signal loss of the reconstructed images compared to reference images, from a computer vision perspective.

The phantom test set consisted of 600 images each of disk phantoms, resolution phantoms, and low-contrast phantoms, resulting in a total of 1800 phantom images. The number of the test set was based on power analysis [18]. We computed the image quality metrics introduced in Section 2.2 to assess the performance of the reconstruction algorithm on phantom data. Two different noise levels were also generated, with SNRs of 12.5 and 25, which resembled the SNRs of a single-coil image in the M4Raw and FastMRI brain dataset respectively, resulting in 3600 phantom images in total.

### 2.6. Experiments

We conducted three main experiments to demonstrate the utility of our image quality assessment approach. Specifically, we evaluated the effect of training and testing data on the performance of the reconstruction networks under the following conditions:

1. Comparison of Fully Sampled vs. Undersampled Training Data:

We trained separate networks using fully sampled k-space data and 2 times undersampled k-space data, described in Section 2.4.3., using the fully sampled inverse Fast Fourier Transform (iFFT) image as reference. The former network was trained to learn the inverse Fast Fourier Transform, while to latter would learn de-aliasing and iFFT together. We denoted them as acceleration factors 1× and 2× in the subsequent figures. This experiment aimed to show differences in reconstruction performance under different sampling conditions.

2. Two Noise Levels for the Testing Phantom:

We evaluated the performance of each trained network using phantom images at different SNR levels: 12.5 and 25.

3. Comparison of 3T vs. 0.3T MR Training Data:

We separately trained the AUTOMAP network using the two datasets, namely M4Raw and FastMRI. This experiment was conducted to assess the impact of magnetic field strength in training data on the reconstruction image quality of the networks.

## 3. Results

### 3.1. Reference iFFT Reconstruction, Fully and UnderSampled AUTOMAP

**Figure 4:**
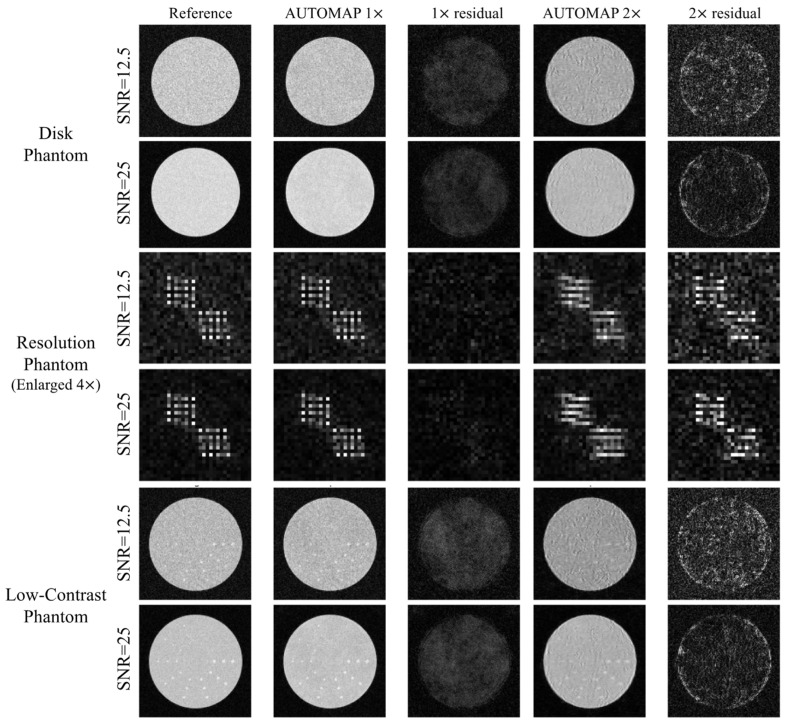
Representative reconstructed phantom images using M4Raw-trained AUTOMAP networks. Reference phantom image (iFFT), AUTOMAP reconstructed images, and the corresponding residuals from fully sampled (AUTOMAP 1×)- and undersampled data (AUTOMAP 2×)-trained networks, for test set SNR levels of 12.5 and 25, are displayed. Resolution phantoms were enlarged 4 times and residual image intensity was multiplied by 5 for better visualization.

In Figure 4, the M4Raw 1× and 1× residual columns demonstrate the three types of phantom images reconstructed with the fully sampled k-space-trained network and the discrepancy between the reconstructed images and the corresponding reference images under the two noise levels. Noticeably, the absolute difference is more pronounced within the phantom region compared to the background region.

**Figure 5:**
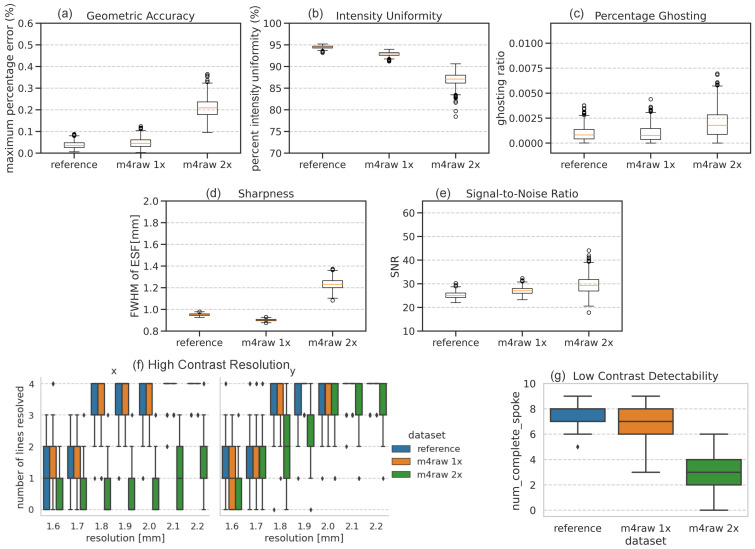
Boxplots of the phantom-based evaluation results across all phantoms for reference image, M4Raw fully sampled k-space-trained network (m4raw 1×), and undersampled k-space-trained network (m4raw 2×): (**a**) geometric accuracy; (**b**) intensity uniformity; (**c**) percentage ghosting; (**d**) sharpness; (**e**) signal-to-noise ratio; (**f**) high-contrast resolution (note that high-contrast resolution in horizontal *x*-axis (left), and vertical *y*-axis (right) are plotted separately); and (**g**) low-contrast detectability. For each boxplot, the center line indicates the median, the box extends from the 1st quartile to the 3rd quartile, the whiskers reach 1.5 times the interquartile range away from the box, and circles indicate outliers.

Figure 5 shows the comparison of evaluated metrics between the M4Raw-trained network and the reference under the noise level of SNR 25. Overall, the geometric accuracy, percentage ghosting, signal-to-noise ratio, resolution, and low-contrast detectability of the reconstructed images closely resemble those of the reference images, indicating the network learned the iFFT process. However, the network reconstructed output exhibits slightly worse intensity uniformity compared to the reference. Moreover, the fully sampled images (M4Raw 1×) display smaller full-width-half-maximum (FWHM) in sharpness measurement, suggesting that image sharpness can be enhanced by the network.

Columns AUTOMAP 2× and 2× residual in Figure 4 illustrate the reconstructed images from the undersampled data-trained network. In comparison to the fully sampled counterparts, the undersampled reconstructions exhibit significantly lower visual quality, with larger residuals. Patterned noise and aliasing artifacts are more prominent, as well as the smeared appearance of the resolution phantom compared to the fully sampled image.

As expected, Figure 5 shows that network trained with undersampled data performs inferiorly to the network trained with fully sampled data in terms of geometric accuracy, intensity uniformity, and low-contrast detectability. Highlights of performance are the significant increase in geometric accuracy percentage error and the expected increases in percentage ghosting and degraded sharpness. Additionally, there is a notable difference in resolution between horizontal and vertical directions, with the horizontal axis exhibiting significantly lower resolution (2.2 mm) due to aliasing comparing to the vertical axis of 1.8 mm resolution. Lastly, the network trained with undersampled data presents a slight increase in SNR, potentially due to the smoothing learned by the network as a result of compensating for k-space undersampling.

### 3.2. Impact of SNR in Test Images

**Figure 6:**
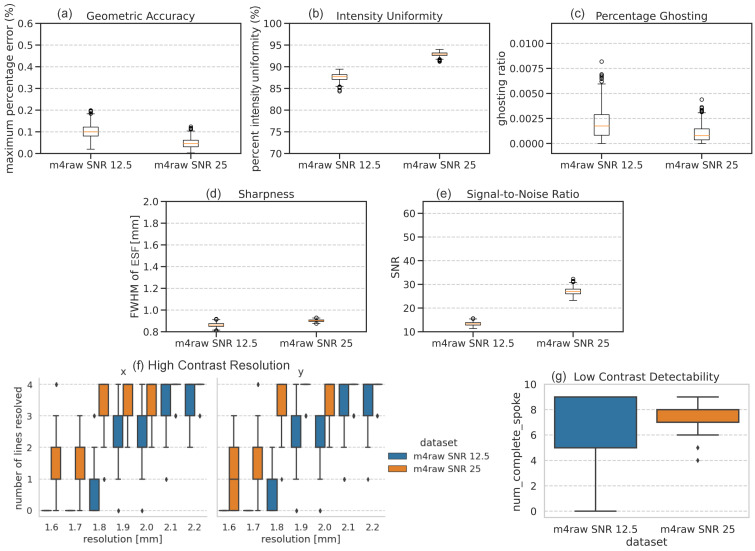
Boxplots of the phantom-based evaluation results across all phantoms reconstructed from the M4Raw fully sampled k-space-trained network at two SNR levels of 12.5 and 25: (**a**) geometric accuracy; (**b**) intensity uniformity; (**c**) percentage ghosting; (**d**) sharpness; (**e**) signal-to-noise ratio; (**f**) high-contrast resolution (note that high-contrast resolution in horizontal *x*-axis (left), and vertical *y*-axis (right) are plotted separately); and (**g**) low-contrast detectability. For each boxplot, the center line indicates the median, the box extends from the 1st quartile to the 3rd quartile, the whiskers reach 1.5 times the interquartile range away from the box, and circles indicate outliers.

The boxplots comparing SNRs in Figure 6 illustrate that the M4Raw network reconstructed image SNR closely matches with the simulated noise levels in the test set, with SNR of 13.4 ± 0.8 and 27.1 ± 1.6 in testing data at SNR levels of 12.5 and 25, respectively. The preservation of SNR again demonstrated that the network trained on M4Raw dataset proficiently learned the iFFT process. However, while SNR remains unaltered, some other image quality metrics degraded with lower SNR levels, including geometric accuracy, intensity uniformity, percentage ghosting, and resolution. In particular, for SNR 12.5 test data, 1.8 mm isotropic resolution can be achieved, while for SNR 25 test data, a resolution of 1.6 mm is observed. As for low-contrast detectability, the variation is smaller in the SNR 25 test set, which suggest low-contrast disks can be more robustly detected with higher SNR. Sharpness remains unaffected by SNR.

### 3.3. Impact of Dataset

**Figure 7:**
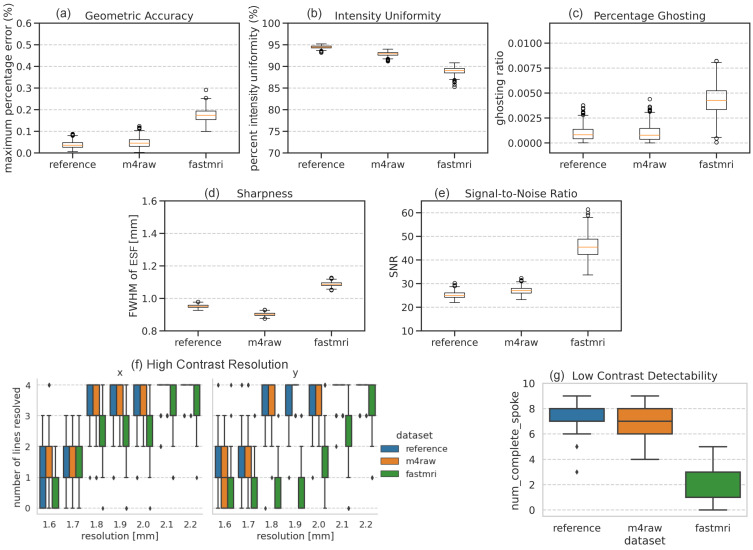
Boxplots of the phantom-based evaluation results across all phantoms for reference image, M4Raw fully sampled k-space-trained network, and FastMRI brain fully sampled k-space-trained network: (**a**) geometric accuracy; (**b**) intensity uniformity; (**c**) percentage ghosting; (**d**) sharpness; (**e**) signal-to-noise ratio; (**f**) high-contrast resolution (note that high-contrast resolution in horizontal x-axis (left), and vertical y-axis (right) are plotted separately; and (**g**) low-contrast detectability. For each boxplot, the center line indicates the median, the box extends from the 1st quartile to the 3rd quartile, the whiskers reach 1.5 times the interquartile range away from the box, and circles indicate outliers.

Figure 7 compares the performance of the networks trained with M4Raw and FastMRI datasets. It reveals that the FastMRI-trained network exhibits lower performance in various image quality metrics, including geometric accuracy, intensity uniformity, ghosting, sharpness, resolution, and low-contrast detectability. Interestingly, SNR is increased in FastMRI-trained networks, indicating SNR does not necessarily follow the trend of other image quality metrics. Images reconstructed by the FastMRI-trained network have different resolutions in the x (horizontal) and y (vertical) dimensions, as demonstrated in the resolution plots, likely due to the stride in our FastMRI data preprocessing where every other k-space data point was selected in the frequency-encoding direction because the original data were oversampled by 2 in this direction.

Detailed evaluation metric values for all three experiments, under two training data undersampling factors, two test set SNRs, and two different sources of training datasets, are summarized in Table 1.

### 3.4. Conventional Metrics

**Figure 8:**
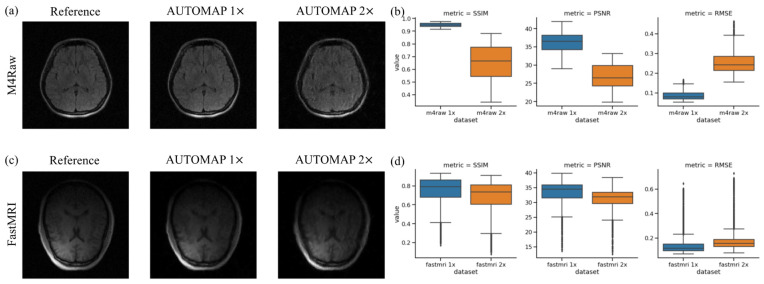
Example reconstructed brain images and conventional evaluation metrics (SSIM, PSNR, and RMSE) for networks trained with fully sampled and undersampled M4Raw and FastMRI datasets. (**a**) Reconstructed images from M4Raw brain test set using the model trained on M4Raw data; (**b**) Boxplots showing SSIM, PSNR, and RMSE results for M4Raw-trained model; (**c**) Reconstructed images from the FastMRI brain test set using the model trained on FastMRI data; (**d**) Boxplots showing SSIM, PSNR, and RMSE results for the FastMRI-trained model. For each boxplot, the center line indicates the median, the box extends from the 1st quartile to the 3rd quartile, the whiskers reach 1.5 times the interquartile range away from the box, and circles indicate outliers.

Figure 8 shows example images reconstructed from the brain test sets and the boxplots of conventional evaluation metric results. In 2× acceleration networks, the brain images appear blurrier, with details and grey and white matter boundaries not clearly visible. Conventional image quality metrics indicate degradation in SSIM, PSNR, and RMSE for the undersampled network, particularly for the M4Raw-trained networks. More outliers are observed in the metrics for the FastMRI-trained network, potentially due to the testing data including images from above the head, consisting only of noise. Overall, the results using these metrics exhibit similar trends to those using the proposed image quality metrics but do not tell the intrinsic image properties. It is important to acknowledge that because the performance of M4Raw-trained and FastMRI-trained networks were evaluated with different datasets, the results are not directly comparable here.

## 4. Discussion

In developing a systematic evaluation pipeline using digital phantoms and automated evaluation metrics for image quality assessment for machine-learning-based MR reconstruction, we obtained several expected and unexpected findings.

As expected, we observed that the AUTOMAP network correctly learned the iFFT relationship and preserved SNR between the reference k-space phantom and the output image. We also noticed an increase in percentage ghosting in the network trained with the undersampled dataset compared to the fully sampled dataset. We expect that this can be explained by increased aliasing artifacts in the undersampling phase-encoding dimension. Additionally, we found that other image quality parameters, such as geometric accuracy, intensity uniformity, sharpness, and resolution, exhibited a decline with increased undersampling or decreased SNR.

However, while most metrics decreased with undersampling, one unexpected discovery was that SNR increased with undersampling. For both the M4Raw and FastMRI dataset-trained models, an inverse trend between SNR metrics and other image quality metrics was also observed. We hypothesize that this is due to the smoothing learned by the network to compensate for k-space undersampling and pure noise input.

These findings underscore several key insights. First, image quality is dependent on multiple factors. For example, SNR may not always align with the trends in other quality metrics. It is possible that SNR improves but resolution degrades due to smoothing. On the other hand, the low-contrast detectability metric, summarizing image resolution and SNR into a single score, better reflects the diagnostic quality in terms of detecting subtle lesions, for example, multiple sclerosis lesions. This emphasizes the need for a multifaceted approach to evaluating image quality in medical imaging depending on the task of interest.

Second, our study revealed that multiple factors, including the training dataset undersampling ratio, testing dataset SNR, the selection of dataset, and the preprocessing techniques, are critical to the machine-learning-based MR reconstruction model. These factors significantly impact the image quality of the reconstructed images and thus require careful consideration and validation during algorithm development.

Furthermore, our digital phantom and evaluation metrics offer a valuable pipeline for fundamental image quality assessment, facilitating the development and optimization of input configurations and network architectures for machine-learning based MRI image reconstruction.

### 4.1. Performance Difference in M4Raw- and FastMRI-Trained Networks

Several reasons may account for the overall better performance of the M4Raw-trained network compared to the FastMRI-trained network. First, in the FastMRI dataset, most of the datasets were acquired with 16 or 20 coils, while the M4Raw dataset had 4 coils. This led to smaller coverage of anatomical regions in each coil of the FastMRI dataset that provided structured information for training.

Second, the FastMRI dataset included slices from above the top of the head, consisting of only noise, while the M4Raw dataset only included slices that were around the center of the brain. The inclusion of pure noise images may have contributed to the lower performance of the FastMRI-trained network. This observation aligns with the finding in the original AUTOMAP paper [2], in which the network trained on pure noise led to less sparse hidden layer activation values that did not utilize spatial correlation information.

Thirdly, due to oversampling in the original k-space in FastMRI data in the frequency-encoding direction, the preprocessing process selected every other k-space data point in this direction. This was designed to match the FOV and image resolution in both directions but could also lead to the unwanted effect of different resolutions in the x- and y-axes in the fully sampled data-trained network.

Lastly, while the M4Raw dataset was acquired at a single center using standardized parameters, the FastMRI dataset was acquired using multiple scanners with different parameters. This difference in acquisition parameters, such as FOV and image contrast, will lead to differences in intrinsic image resolution and SNR within the FastMRI dataset. We expect that non-uniformity in acquisition parameters may lower overall network performance but might increase generalizability, which can be further investigated in future work.

### 4.2. Limitations

We acknowledge that our study has some limitations. First, we only tested our evaluation methods on one end-to-end MRI reconstruction network. Our goal was to demonstrate the applicability of our image quality evaluation pipeline, not to evaluate the performance of any particular reconstruction network, but we recognize that benchmarking across multiple networks, including a denoising network, de-aliasing network, hybrid network, etc., would have strengthened our approach.

Additionally, while our approach separated images from each coil in training to increase the total number of inputs, coil sensitivity may impact the performance of the network. In this study, our network was trained to learn the simple iFFT reconstruction and this would pose minimal effect. However, for more advanced reconstructions, coil combined input and output are preferred. This will reduce the total number of the training set, but data augmentation may help resolve the data size limit.

### 4.3. Future Work

In future work, validating our method on other reconstruction networks can provide valuable insight for image quality analysis across networks and demonstrate the broader applicability of our approach. Some promising candidates for validation include DC-CNN [3] for image space denoising, DAGAN [28] for anti-aliasing, VarNet [29] for multicoil input, and KIKI-net [30] for hybrid cross-domain enhancement. By testing our method on diverse networks, we can assess its effectiveness across various reconstruction scenarios and potentially guide the development and improvement of machine-learning-based MR image reconstruction.

Another important area for future research is to compare our metrics with those obtained from human or model observers, similar to what has been explored in [31,32,33]. By aligning quantitative metrics with human perception, we can better understand the strengths and limitations of our approach and ensure that it accurately captures clinically relevant aspects of image quality. This validation process will enhance the credibility and reliability of our evaluation framework in replacing human observers in the image quality evaluation process.

## 5. Conclusions

In conclusion, we developed a systematic evaluation pipeline for image quality assessment by creating digital phantoms and using automated approaches to measure multiple metrics for ML-based MR reconstruction following ACR phantom test guidance. We demonstrated its effectiveness by evaluating an example AUTOMAP reconstruction network across multiple training and testing scenarios. The performance results indicate that training data acquired with a lower undersampling factor and coils of larger anatomical coverage are preferred to achieve a better performing reconstruction model. This evaluation pipeline can contribute to standardized evaluation of reconstruction algorithms and assist in optimization of ML-based MR reconstruction networks. Future work includes applying our image quality evaluation pipeline to other types of reconstruction networks and validating automatically calculated metrics against human observations.

## Figures and Tables

**Table 1:** Summary of mean and standard deviation of all image metric values for AUTOMAP networks trained with M4Raw 1×, M4Raw 2×, FastMRI 1×, and FastMRI 2× data, and the reference reconstruction (iFFT).

Training Set	Test Set SNR	Geometric Accuracy (%)	Intensity Uniformity (%)	Ghosting Ratio *	Sharpness [mm]	SNR	Low-Contrast Detectability
M4Raw 1×	12.5	0.10 ± 0.03	87.6 ± 0.8	0.002 ± 0.002	0.86 ± 0.02 **	13.4 ± 0.8	**7.0 ± 2.6**
	25	**0.05 ± 0.02**	**92.9 ± 0.5**	**0.001 ± 0.001**	**0.90 ± 0.01**	27.1 ± 1.6	**7.1 ± 0.9**
M4Raw 2×	12.5	0.29 ± 0.06	80.6 ± 2.2	0.003 ± 0.002	1.26 ± 0.06	14.4 ± 1.8	3.6 ± 2.5
	25	0.21 ± 0.05	87.0 ± 1.5	0.002 ± 0.001	1.23 ± 0.05	29.5 ± 4.0	3.3 ± 1.3
FastMRI 1×	12.5	0.21 ± 0.03	83.1 ± 1.3	0.005 ± 0.003	1.10 ± 0.02	19.7 ± 2.1	1.5 ± 1.7
	25	0.17 ± 0.03	89.0 ± 0.8	0.004 ± 0.001	1.09 ± 0.01	45.7 ± 4.7	1.4 ± 1.2
FastMRI 2×	12.5	0.37 ± 0.07	79.1 ± 2.2	0.004 ± 0.003	1.65 ± 0.06	23.2 ± 3.4	1.2 ± 1.2
	25	0.32 ± 0.05	85.1 ± 1.5	0.004 ± 0.002	1.64 ± 0.05	**48.1 ± 7.2**	0.8 ± 0.6
Reference (iFFT)	12.5	0.09 ± 0.03	88.9 ± 0.7	0.002 ± 0.001	0.91 ± 0.02	12.6 ± 0.7	8.0 ± 1.9
	25	0.04 ± 0.02	94.5 ± 0.3	0.001 ± 0.001	0.95 ± 0.01	25.2 ± 1.4	7.6 ± 0.7

* Metrics are unitless unless specified. ** Best performance(s) by ML-based reconstructions are highlighted in bold.

## Data Availability

The M4Raw dataset presented in the study is openly available in Zenodo at https://zenodo.org/records/8056074 (accessed on 2 October 2023), and the FastMRI dataset is available at https://fastmri.med.nyu.edu/ (accessed on 27 September 2023). The AUTOMAP reconstruction network is available on GitHub at https://github.com/MattRosenLab/AUTOMAP (accessed on 11 October 2023). The original phantom creation and evaluation code presented in the study is openly available on GitHub at https://github.com/DIDSR/mr-recon-eval-core (accessed on 25 April 2024).

## Disclaimer

The mention of commercial products, their sources, or their use in connection with material reported herein is not to be construed as either an actual or implied endorsement of such products by the Department of Health and Human Services. This is a contribution of the U.S. Food and Drug Administration and is not subject to copyright.
